# Cross-Domain Statistical–Sequential Dependencies Are Difficult to Learn

**DOI:** 10.3389/fpsyg.2016.00250

**Published:** 2016-02-25

**Authors:** Anne M. Walk, Christopher M. Conway

**Affiliations:** ^1^Neurocognitive Kinesiology Lab, Department of Kinesiology and Community Health, University of Illinois at Urbana-Champaign, UrbanaIL, USA; ^2^NeuroLearn Lab, Department of Psychology, Georgia State University, AtlantaGA, USA

**Keywords:** statistical learning, implicit learning, sequential learning, cross-modal learning, multisensory integration, modality constraints, artificial grammar learning

## Abstract

Recent studies have demonstrated participants’ ability to learn cross-modal associations during statistical learning tasks. However, these studies are all similar in that the cross-modal associations to be learned occur simultaneously, rather than sequentially. In addition, the majority of these studies focused on learning across sensory modalities but not across perceptual categories. To test both cross-modal and cross-categorical learning of sequential dependencies, we used an artificial grammar learning task consisting of a serial stream of auditory and/or visual stimuli containing both within- and cross-domain dependencies. Experiment 1 examined within-modal and cross-modal learning across two sensory modalities (audition and vision). Experiment 2 investigated within-categorical and cross-categorical learning across two perceptual categories within the same sensory modality (e.g., shape and color; tones and non-words). Our results indicated that individuals demonstrated learning of the within-modal and within-categorical but not the cross-modal or cross-categorical dependencies. These results stand in contrast to the previous demonstrations of cross-modal statistical learning, and highlight the presence of modality constraints that limit the effectiveness of learning in a multimodal environment.

## Introduction

Many organisms have the ability to detect invariant patterns and associations from a seemingly chaotic environment. One such ability, statistical–sequential learning, involves the learning of statistical patterns across items presented in sequence (Saffran et al., 1996; Daltrozzo and Conway, 2014). Statistical learning appears to be central to the development of many cognitive functions, especially language (Saffran et al., 1996; Conway et al., 2010; Misyak et al., 2010; Nemeth et al., 2011; Arciuli and Simpson, 2012; Kidd, 2012; Misyak and Christiansen, 2012). Traditionally, statistical learning has been studied in a unimodal manner, presenting participants with stimuli to a single sensory modality, such as audition, vision, or touch (Saffran et al., 1996; Fiser and Aslin, 2001; Kirkham et al., 2002; Conway and Christiansen, 2005). However, in many natural circumstances, such as spoken language, multiple sensory modalities are involved. For example, sighted individuals make extensive use of visual facial information, such as the movement of the mouth, to aid in speech perception (Rosenblum, 2008).

Despite the importance of multisensory integration in language processing and other areas of cognition, only recently has multisensory integration been investigated in the context of statistical learning. Toward this end, Mitchel and Weiss (2011) presented unimodal auditory and visual input streams simultaneously to participants and manipulated the audiovisual correspondence across the two modalities. They found that learners could extract the statistical associations in both input streams independently of the other (consistent with the findings of Seitz et al., 2007) except when the triplet boundaries were desynchronized across the visual and auditory streams. In such conditions, learning was disrupted, suggesting that statistical learning is affected by cross-modal contingencies. Other studies have similarly shown that input presented in one modality can affect learning in a second concurrently presented modality. For instance, Cunillera et al. (2010) showed that simultaneous visual information could improve auditory statistical learning if the visual cues were presented near transition boundaries (see also Robinson and Sloutsky, 2007; Sell and Kaschak, 2009; Mitchel and Weiss, 2010; Thiessen, 2010). More recently, Mitchel et al. (2014) used the McGurk illusion to demonstrate that learners can integrate auditory and visual input during a statistical learning task, suggesting that statistical computations can be performed on an integrated multimodal representation.

Although these studies are all clear demonstrations of multimodal integration during statistical learning tasks, they use concurrent auditory and visual input. That is, the visual and auditory inputs were presented simultaneously, and learners were tested on their ability to learn these simultaneous cross-modal associations. No studies to our knowledge have tested the extent that cross-modal statistical associations can be learned and integrated across time as elements in a sequence, in which an auditory stimulus (e.g., a tone) might be associated with the next occurrence of a particular visual stimulus (e.g., a shape) or vice-versa. In addition, previous studies have used multi-sensory patterns containing cross-modal regularities across sensory modalities, but none to our knowledge have tested learning of dependencies across different perceptual categories but that are within the same sensory modality (i.e., color and shape or tones and non-words). It is possible that learning cross-modal dependencies may have different computational demands than the learning of cross-categorical dependencies, perhaps due to differences in perceptual or attentional requirements.

The aim of the present study, therefore, was to investigate the limits of cross-domain statistical–sequential learning. From a purely associative learning framework, it might be hypothesized that statistical patterns should be learned just as readily between stimuli regardless of their modality or perceptual characteristics (i.e., learning a dependency between items A and B should not be any different than learning a dependency between items A and C). Such an unconstrained view of statistical learning was common in its early formulations (see Frensch and Runger, 2003 and Conway et al., 2007 for discussion). However, it is now known that statistical learning is constrained by attentional and perceptual factors. For example, statistical learning of non-adjacent relationships is heavily influenced by perceptual similarity, with learning improving when the non-adjacent elements are perceptually similar to one another (i.e., have a similar pitch range or share some other perceptual cue; Creel et al., 2004; Newport and Aslin, 2004; Gebhart et al., 2009). Likewise, Conway and Christiansen (2006) proposed that statistical learning is analogous to perceptual priming, in which networks of neurons in modality-specific brain regions show decreased activity when processing other items within the same modality that have similar underlying regularities or structure (see also, Reber et al., 1998; Chang and Knowlton, 2004; Conway et al., 2007). Recent neuroimaging evidence confirms that statistical learning is mediated at least in part by processing in unimodal, modality-specific brain regions (Turk-Browne et al., 2009) – in addition to involving “downstream” brain regions that appear less tied to a specific perceptual modality including Broca’s area, the basal ganglia, and the hippocampus (Lieberman et al., 2004; Opitz and Friederici, 2004; Petersson et al., 2004; Abla and Okanoya, 2008; Karuza et al., 2013; Schapiro et al., 2014). Thus the existing literature suggests that statistical learning involves a combination of bottom-up perceptual processing via unimodal, modality-specific mechanisms, but also more domain-general learning and integration processes that perhaps occur further downstream (Keele et al., 2003; Conway and Pisoni, 2008; Daltrozzo and Conway, 2014; Frost et al., 2015).

Thus, the learning of sequential patterns appears to be at least partly constrained by the nature of the sensory and perceptual processes that are engaged. Another way to think of this is that statistical learning is likely influenced by Gestalt-like principles that make it easier to learn associations between items in the same modality or that share perceptual features (Newport and Aslin, 2004). Consequently, statistical learning of cross-modal or cross-categorical sequential associations might be more challenging than the previous empirical research seems to indicate. It is possible that the previous demonstrations of multisensory integration during statistical learning tasks that used concurrent auditory and visual input (e.g., Cunillera et al., 2010; Mitchel and Weiss, 2011; Mitchel et al., 2014) were less cognitively demanding than learning elements across a temporal sequence. It is currently an open question to what extent statistical–*sequential* cross-modal and cross-categorical dependencies can be learned.

**FIGURE 1:**
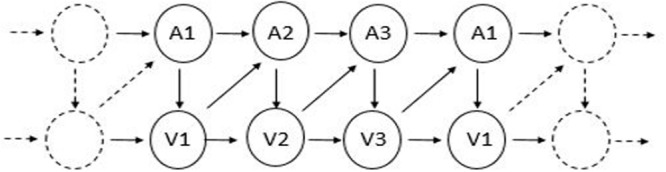
**The artificial grammar used in both Experiments.** “V” and “A” refer to visual and auditory stimuli, respectively.

To test cross-modal and cross-categorical statistical learning, we employed an artificial grammar learning (AGL) paradigm, commonly used to study implicit and statistical learning (Seger, 1994; Perruchet and Pacton, 2006), in which stimuli are determined by a finite state grammar. Unlike previous statistical learning or AGL tasks, our paradigm used a series of inputs from different sensory modalities and/or perceptual categories, with each individual unit presented in succession. In this manner, we could test whether participants can learn cross-domain dependencies across the temporal sequence. The grammar itself (see **Figure 1**), created by Jamieson and Mewhort (2005) and also used by Conway et al. (2010), has certain advantages over other artificial grammars commonly used. First, unlike most other grammars including the classic “Reber” grammar (Reber, 1967) and countless others, there are no positional constraints. That is, each element of the grammar can occur at any position, with equal frequency, preventing position information – such as which elements or pairs of elements occur at the beginning versus the ending of sequences – from becoming a confound. Second, there are also no constraints on sequence length. A large set of stimuli can be generated at a particular length (such as length 6 used in the present study), preventing sequence length from becoming a confound. Finally, the grammar describes the probability in which a successive element (n+1) can occur given the previous element (*n*). This means that primarily first-order element transitions are contained in the grammar; thus, “learning the grammar” in this case generally means one thing: learning the forward-transition, adjacent element statistics^1^, making interpretation about what is learned or not learned relatively straightforward. Consequently, this also makes it easy to design sequences containing both cross-domain and within-domain dependencies.

In Experiment 1, participants were exposed to input sequences generated from the artificial grammar that were composed of tones interspersed with pictures of shapes. Importantly, the sequences consisted of both within-modal (e.g., tone–tone or shape–shape) and cross-modal associations (e.g., tone–shape or shape–tone). In Experiment 2 the sequences were composed of stimuli from two different perceptual categories within the same sensory modality (shapes and colors for the visual stimuli and tones and single syllable non-words for the auditory stimuli), allowing us to test cross-categorical learning. By incorporating a combination of within- and cross-modality stimuli (Experiment 1) and within- and cross-category stimuli (Experiment 2), we were able to examine to what extent participants naturally learn statistical–sequential patterns across sensory domains and perceptual categories.

## Experiment 1: Learning Across Sensory Modalities

### Materials and Methods

#### Participants

Fifteen undergraduate students from a Midwest university participated (Age Range = 18–23; Mean Age = 18.93; Females = 9). All were fluent English speakers. All participants were enrolled in college at the time of their participation. Participants received credit toward partial fulfillment of an undergraduate course as compensation for their time. The study was carried out in accordance with the recommendations of the Saint Louis University Institutional Review Board. All participants gave written informed consent in accordance with the Declaration of Helsinki.

#### Stimulus Materials

We used an artificial grammar consisting of three visual and three auditory elements. The visual elements were abstract black and white shapes used previously in a study by Joseph et al. (2005) and considered difficult to verbally label. The auditory elements were three pure tones that were generated using Audacity software, having frequencies of 210, 286, and 389 Hz, which neither conform to standard musical notes nor have standard musical intervals between them (as used in Conway and Christiansen, 2005).

**FIGURE 2:**
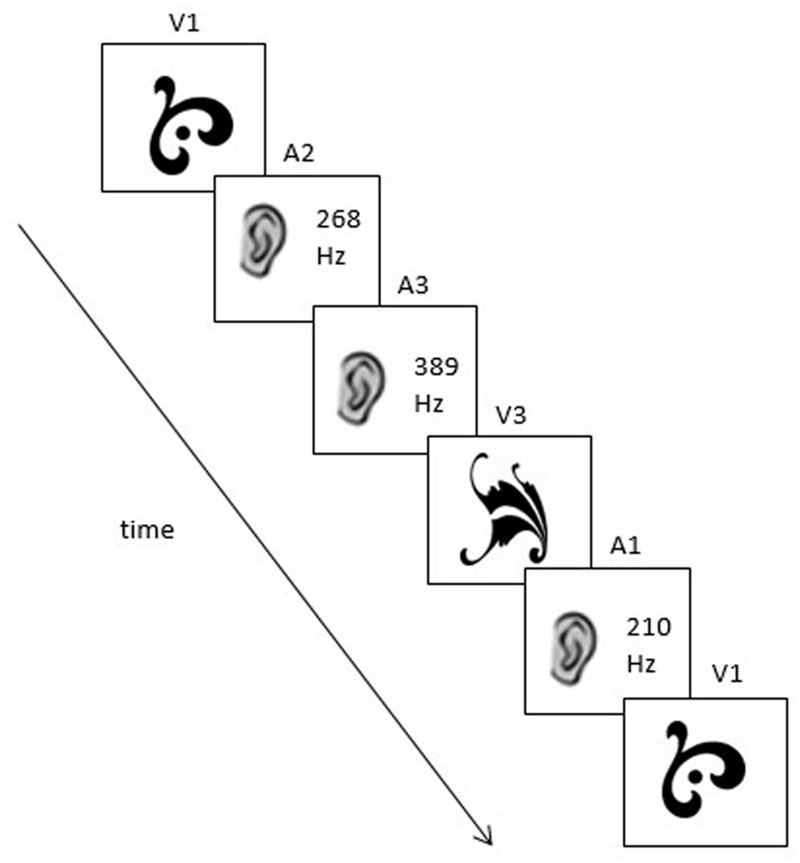
**A possible grammatical sequence used in Experiment 1 (V1–A2–A3–V3–A1–V1)**.

Each sequence was generated by an artificial grammar with constrained probabilities (similar to those used in Jamieson and Mewhort, 2005 and Conway et al., 2010; See **Figure 1**). The grammar dictates that any given element can be followed by one element from the same sensory modality and one element from the other sensory modality. For example, if V1 is the starting element, it can be followed by either A2 or V2 with an equal probability (50/50%). Thus, V1–A2–A3–V3–A1–V1 is an example of a sequence that could be generated by this grammar; it contains four cross-modal dependencies (V1–A2; A3–V3; V3–A1; A1–V1) and one within-modal dependency (A2–A3; see **Figure 2**).

Using the grammar presented in **Figure 1**, a single “learning” stream was generated and used for all participants, consisting of 180 stimuli presented in sequence. In addition, three types of six-item test sequences were constructed: grammatical sequences, ungrammatical sequences containing within-modal violations, and ungrammatical sequences containing cross-modal violations. To create within-modal violation sequences, all within-modal dependencies were altered so that they violated the grammar, with the cross-modal dependencies remaining grammatical. For cross-modal violation sequences, all cross-modal dependences were altered so that they violated the grammar, with the within-modal dependencies remaining grammatical. For example, in the case of a within-modal violation sequence, if the grammatical sequence was V1–A2–A3, the element A3 would be replaced with the other auditory element, so that the sequence would become V1–A2–A1. From that point, the grammar would be renewed and would continue correctly until another within-modal transition occurred. We constructed 20 grammatical test sequences, 10 within-modal ungrammatical test sequences, and 10 cross-modal ungrammatical test sequences. The total number of violations in the within-modal violation stimulus set (28 violations total or 2.8 violations on average per sequence) and cross-modal violation stimulus set (25 violations total or 2.5 violations on average per sequence) were roughly equal and not statistically different from each other (*t* = 0.669, *p* = 0.512). All test sequences are listed in the Appendix (Table A1).

#### Procedure

All participants completed a learning phase and a test phase. In the learning phase, participants were seated in front of a computer monitor with a pair of headphones. They were instructed to pay attention to the pictures and sounds that were displayed. Participants were exposed to the continuous stream of 180 shapes and tones that was generated using the grammar. The durations for both the auditory and visual stimuli were 1000 ms each, with an ISI of 1000 ms, giving a total learning phase duration of 6 min.

In the test phase of the experiment, participants were told that the input stream they had observed was created according to certain rules that determined the order that each element was presented. Participants were then presented with each of the six-item test sequences and were asked to determine if each item “followed the rules” (i.e., was grammatical) or “did not follow the rules” (i.e., was ungrammatical). Participants responded by pressing one of two buttons to indicate their choice. Participants were exposed to the novel grammatical, within-modal ungrammatical, and cross-modal ungrammatical sequences in random order. Within each test sequence, the stimulus durations (1000 ms) and ISI (1000 ms) were the same as used in the learning phase. Participants had as much time as needed to make their response, after which the next test trial began. Note that for both the learning and test phases the auditory and visual tokens were randomly assigned and mapped to the elements of the grammar. For one participant A1 might be the 210 Hz tone, but for another participant A1 might be the 286 Hz tone, etc. Thus, even though each participant received the same learning and test items in terms of their underlying structural patterns, the actual tokens that mapped onto these patterns differed for each participant, determined randomly.

### Results and Discussion

Results are shown in **Table 1**, displaying the percentage of test items classified correctly for each test item type. Performance on the within-modal violation sequences was numerically the highest (*M* = 64.00%) followed by performance on the grammatical sequences (*M* = 60.67%), and lastly the cross-modal violations (*M =* 48.67%). To explore the accuracy of participants’ performance on the three item types, a repeated measures analysis of variance (ANOVA) was conducted, indicating a significant main effect of sequence type [*F*(2,28) = 4.893, *p* = 0.015, $\eta_{p}^{2}$ = 0.259]. A test of simple comparisons with a Bonferroni correction indicated that there was a statistically significant difference between performance on the within-modal violations and the cross-modal violations (*p* < 0.05). A series of single sample t-tests was run to test the average performance on each item type to chance (50%). The analysis indicated that participants performed significantly above chance on the grammatical items (*t* = 4.00, *p* ≤ 0.05, Cohen’s *d* = 1.04) and the within-modal ungrammatical items (*t* = 4.18, *p* ≤ 0.001, *d* = 1.08), but not on the cross-modal ungrammatical items (*t* = –0.31, *p* ≥ 0.10, *d* = 0.08).^2^

**Table 1:** Mean Performance (percent correct) and standard deviations for Experiments 1 and 2.

	Experiment 1 (Within- vs. cross-modal)	Experiment 2 (Within- vs. cross-category)	
		Visual	Auditory
Grammatical	60.67 (16.85)	51.88 (17.11)	60.63 (11.81)
Within-modal/category	64.00 (12.98)	65.00 (18.97)	80.63 (15.69)
Cross-modal/category	48.67 (10.33)	51.25 (21.25)	50.63 (19.14)

The findings from Experiment 1 indicate that participants were more proficient at detecting within-modal violations – that is, violations occurring between stimuli in the same sensory modality – than at detecting cross-modal violations that occurred between stimuli in different modalities. In fact, participants were completely unable to successfully detect violations of cross-modal contingencies. The lack of cross-modal learning stands in contrast to previous studies of multimodal statistical learning, which differed from the present study by their use of simultaneous rather than sequential cross-modal dependencies.

## Experiment 2: Learning Across Perceptual Categories Within A Single Sensory Modality

The results of Experiment 1 demonstrate that when exposed to multimodal sequential patterns, within-modal but not cross-modal violations can be detected. In Experiment 1, the cross-modal associations were multisensory (i.e., consisting of audio–visual or visual–audio links). Another way to probe multimodal learning is to test the ability to learn associations that are within the same sensory modality (e.g., vision or audition) but that exist between different perceptual categories (e.g., tones and words or colors and shapes).

### Materials and Methods

#### Participants

A new group of 32 undergraduate students from the same Midwestern University participated in the study (Age Range = 18–22; Mean Age = 19.54, 1 not reported; Females = 20, 2 not reported). All students were fluent speakers of English. All participants were enrolled in college at the time of their participation and received credit toward partial fulfillment of an undergraduate course as compensation for their time. Participants were randomly assigned to one of two conditions, auditory or visual. The study was carried out in accordance with the recommendations of the Saint Louis University Institutional Review Board. All participants gave written informed consent in accordance with the Declaration of Helsinki.

#### Stimulus Materials

For Experiment 2, four types of stimuli were used: the same black and white shapes and same pure tones as before, as well as three colored circles (red, green, and blue) and three single-syllable non-words (“dak,” “pel,” and “vot”). This provided us with four sets of perceptual categories, two sets (tones, non-words) for the auditory modality and another two sets (shapes, colors) for the visual modality.

**FIGURE 3:**
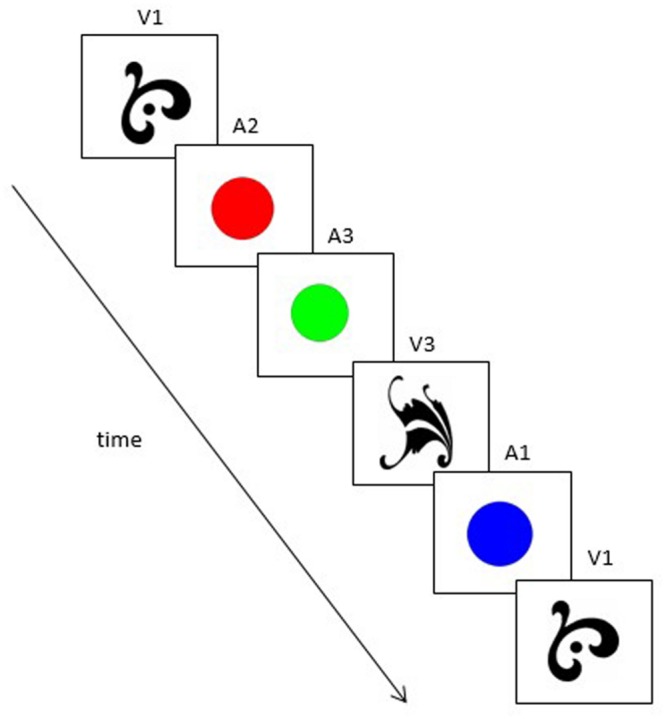
**An example of a grammatical sequence used in the visual condition of Experiment 2 (V1–A2–A3–V3–A1–V1).** Note that in this case, “A” no longer refers to an auditory stimulus but to the second category of visual stimuli (colors).

**FIGURE 4:**
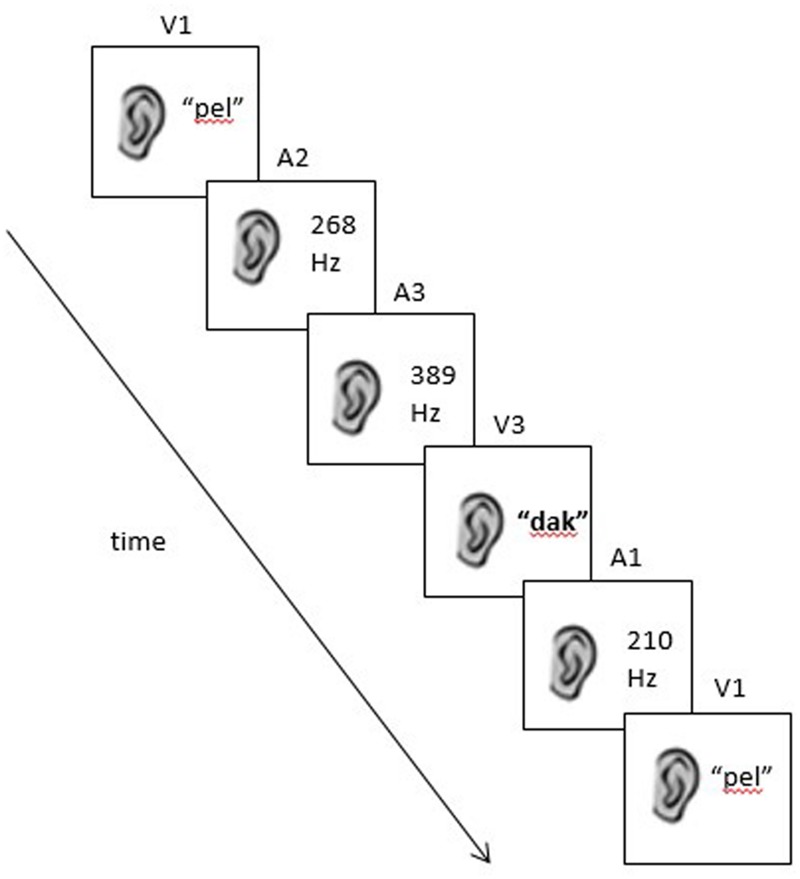
**An example of a grammatical sequence used in the auditory condition dof Experiment 2 (V1–A2–A3–V3–A1–V1).** Note that in this case, “V” no longer refers to a visual stimulus but to the second category of auditory stimuli (non-words).

The learning stream and test sequences were the same as used in Experiment 1, except that the inputs were altered to reflect the two new stimulus sets. In the visual condition, A and V elements of the original grammar were replaced with shapes and colors (see **Figure 3**). In the auditory condition, A and V elements of the grammar were replaced with tones and non-words (see **Figure 4**). Mirroring the design of Experiment 1, the new stimuli formed a set of grammatical sequences, within-categorical ungrammatical sequences, and cross-categorical ungrammatical sequences.

#### Procedure

The procedure was the same as in Experiment 1, except that participants were assigned to one of two groups (auditory or visual). Participants assigned to the auditory group received input sequences composed of the two categories of auditory stimuli (pure tones and non-words), while participants assigned to the visual condition received sequences composed of the two categories of visual stimuli (abstract shapes and colored circles).

### Results and Discussion

As in Experiment 1, the accuracy scores are displayed as percentages (**Table 1**). To investigate the performance of the two groups, a repeated measures, mixed factor ANOVA was conducted with group (visual and auditory) as the between subjects variable and sequence type (grammatical, within-category violations, and cross-category violations) as the within subjects variable. The results of the Mauchly’s test of sphericity suggested that the sphericity assumption of a repeated measures ANOVA was violated [Mauchly’s *W* = 0.508. *p* ≤ 0.001]. Therefore, in all subsequent analyses, the Greenhouse–Geisser test of the multivariate analysis is reported. The analysis revealed significant main effects of sequence type [*F*(1.34,40.20) = 13.56, *p* ≤ 0.001, $\eta_{p}^{2}$ = 0.311] and group [*F*(1,30) = 4.794, *p* ≤ 0.05, $\eta_{p}^{2}$ = 0.138]. A test of simple comparisons with a Bonferroni correction was run to further explore the main effects. It revealed that participants across the two groups performed significantly higher on the within-category violation sequences than either the grammatical sequences (*p* ≤ 0.001) or the cross-category violation sequences (*p* ≤ 0.01). Across sequence type, participants in the auditory condition performed better than participants in the visual condition.

A series of independent samples *t*-tests were run on each group of participants comparing performance to chance for each sequence type. The analysis showed that participants in the visual group only had better than chance performance for the within-category items (*t* = 3.162, *p* ≤ 0.01, *d* = 0.79). However, participants in the auditory group showed better than chance performance for both the within-category violation items (*t* = 7.806, *p* ≤ 0.001, *d* = 1.95) and the grammatical items (*t* = 3.597, *p* ≤ 0.01, *d* = 0.90). Performance for cross-category items was at chance levels for both groups.^3^

The pattern of results mirrors that seen in Experiment 1 but extends it to the learning of statistical patterns across perceptual categories within the same sensory modality. Participants were able to detect statistical–sequential violations within the same perceptual category but were unable to detect violations across perceptual categories. Furthermore, a modality effect was observed, consistent with previous research showing that audition displays higher levels of learning for sequentially presented patterns (Conway and Christiansen, 2005; Emberson et al., 2011).

## General Discussion

The findings from this study suggest that learning statistical–sequential associations within a perceptual or sensory domain is easier than learning across domains. In Experiment 1, participants displayed significantly higher accuracy for identifying sequential violations that occurred between elements within the same sensory modality (e.g., tone–tone or shape–shape) than they did identifying violations at cross-modal boundaries (e.g., tone–shape). Likewise, participants in Experiment 2 showed significantly better performance identifying violations between elements in the same perceptual category (e.g., tone–tone, word–word, shape–shape, or color–color) than they did identifying violations at category boundaries within the same sensory modality (e.g., tone–word or color–shape). It appears that statistical learning is biased to operate first within a particular perceptual category, before integrating items across categories or across modalities.

The test sequences were constructed such that some items contained within-domain violations of the grammar whereas other items contained cross-domain violations. These violations were all adjacent dependencies. However, the grammar also contains subtle non-adjacent regularities that participants possibly could have learned. The statistical strength of these non-adjacent dependencies, however, is relatively weak compared to the strength of the adjacent-item dependencies, with transitional probabilities being 0.33 for the former and 0.5 for the latter (that is, the grammar stipulates two adjacent-item links for each stimulus and three non-adjacent item links for each stimulus). Because Gómez (2002) demonstrated that the learning of non-adjacent dependencies occurs only when the adjacent-item statistics are unreliable, which was not the case in the present study, it is more likely that participants’ performance was based primarily on the learning of adjacent-item dependencies. Regardless, even if some amount of non-adjacent item statistics were learned, it does not much change the overall finding of this study, which is that cross-domain sequential dependencies appear to be difficult to learn.

These findings furthermore provide evidence that is not entirely consistent with a purely domain-general view of statistical learning (Altmann et al., 1995; Manza and Reber, 1997; Kirkham et al., 2002), in which all dependencies would be expected to be treated the same and learned at comparable levels. Instead, the present findings are consistent with previous research suggesting the presence of modality constraints and perceptual grouping principles affecting statistical learning (Creel et al., 2004; Conway and Christiansen, 2005, 2006; Gebhart et al., 2009; Emberson et al., 2011). Although learning across domains may operate via similar computational principles, it has been argued that there may exists a distributed network of modality-constrained learning mechanisms (Conway and Christiansen, 2005; Frost et al., 2015). Under this view, learning associations between stimuli taking on the same sensory and perceptual characteristics takes precedence over the learning of associations across perceptual categories and modalities. Furthermore, previous evidence suggests that each sensory modality computes statistical associations for its particular input type only, with patterns learned through one sensory modality or perceptual category staying representationally bound to that particular domain (Conway and Christiansen, 2006). However, likely due to differences in attentional or memory requirements, cross-modal statistical learning appears to be possible when the cross-modal contingencies occur together in time, rather than across a temporal sequence as was the case in the present study (Seitz et al., 2007; Sell and Kaschak, 2009; Cunillera et al., 2010; Thiessen, 2010; Mitchel and Weiss, 2011; Mitchel et al., 2014).

This type of hierarchical arrangement for statistical learning perhaps is not surprising given what we know about basic brain organization. In general, hierarchically lower level (i.e., upstream) brain areas mediate the processing of specific stimulus properties (e.g., color, motion, pitch, etc.) whereas at increasingly hierarchically higher (i.e., downstream) brain areas, more abstract and multimodal properties are integrated (e.g., speech, complex visual objects, etc.). Although certainly perception is not entirely modular, with downstream “multimodal” regions able to influence upstream areas through feedback connections (e.g., Driver and Spence, 2000), it is clear that both segregation (at upstream levels) and integration (at downstream levels) are foundational aspects of brain organization and processing (Friston et al., 1995). From a neurobiological standpoint, it is likely that statistical–sequential learning recapitulates these general principles of segregation and integration in the brain.

Although the present study found no evidence for multimodal integration of cross-modal sequential dependencies, this does not mean that it cannot occur under different experimental conditions, for example with a longer learning phase duration or manipulations to promote attention to the cross-modal dependencies. In fact, a prominent theory of sequence learning, based upon neuroimaging and behavioral findings using the serial reaction time task, posits the existence of two partially dissociable neurocognitive learning mechanisms: an implicit unidimensional system that operates over inputs within the same perceptual modality, and a multidimensional system that operates over inputs across perceptual modalities or categories (Keele et al., 2003). Importantly, the latter system appears to require attentional resources to learn the cross-modal or cross-categorical associations (see Daltrozzo and Conway, 2014, for a similar two-system view of statistical learning). Applying such a dual-system perspective to the present findings would seem to indicate that only the implicit unidimensional system was active during this task, not the multidimensional system, presumably due to a lack of attentional focus on the cross-domain dependencies. Regardless of whether one adopts the dual-system view, it appears that even if multimodal sequential integration is possible, it is not the initial gateway to learning under standard incidental learning conditions as used in the present study. Instead, input modalities during statistical learning appear to be initially percept specific, and perhaps only become integrated at subsequent levels of processing when additional cognitive resources are deployed.

Finally, it could be argued that the manner in which multisensory statistical learning was tested, with cross-modal dependencies occurring across a temporal sequence, is not ecologically realistic of what humans or other complex organisms typically encounter in the world. To this point, we offer two considerations. First, in the present study we aimed to create a learning situation that would probe the limits of multisensory statistical learning across a temporal sequence. Even if the patterns presented to participants are not ecologically realistic, the findings provide insight about possible limitations constraining cross-domain statistical learning and provide hints to the underlying architecture of the learning mechanisms themselves. Second, the issue of ecological validity implies a certain chain of reasoning: that humans have difficulty learning cross-domain sequential patterns because they are not exposed to such patterns in the world. On the other hand, the direction of causality could in fact go in the other direction: perhaps the reason why we observe minimal cross-domain sequential dependencies in our environments is precisely because of the limitations inherent in our learning faculties. For instance, it is conceivable that natural language could have evolved to capitalize on cross-domain sequential dependencies (e.g., it is logically possible that sentences could be composed not just of sequences of auditory–vocal units, but sequences of spoken words interleaved with hand and arm gestures). The lack of such cross-domain sequential dependencies in human communication could be due to the inability of humans to effectively learn such sequential patterns, consistent with the view that natural language has evolved to adapt to the processing constraints and limitations of the human brain (Christiansen and Chater, 2008).

In sum, the results of these two experiments show that when statistical–sequential input is composed of elements from two different perceptual categories or sensory modalities, participants can detect violations that occur between elements within a single domain, but not violations that occur between domains. These findings stand in contrast to previous demonstrations of cross-modal statistical learning and provide new insights about the difficulties facing learners exposed to complex multisensory environments.

## Author Contributions

AW, CC both contributed to the conception and design of this work and interpretation of data; the drafting and revising of the manuscript; and final approval of the version to be published. AW furthermore carried out the acquisition and analysis of the data.

## Conflict of Interest Statement

The authors declare that the research was conducted in the absence of any commercial or financial relationships that could be construed as a potential conflict of interest.

## Footnotes

1 As pointed out by one of the reviewers of the current manuscript, non-adjacent dependencies are also contained in the grammar that could possibly be learned by participants. Each element in the grammar has two within-domain non-adjacent dependencies and one cross-domain non-adjacent dependency, with each following with equal probability (0.33 each). For example, A1 can be followed by A3 (with an intervening A2), V2 (with an intervening V1 or A2), or A2 (with an intervening V1). To what extent these non-adjacent dependencies impact performance will be discussed in more detail in the Section “Discussion.”

2 Due to a design error, two within-modal violation test items (#25 and #26) were found to be problematic. Instead of all within-modal transitions being ungrammatical, each had one within-modal dependency that was not violated. In addition, these two items began with a repetition of a single stimulus, which could add unwanted salience to those items. Thus, all statistical analyses were rerun after taking out participant responses to these two items. The overall pattern of results remained similar: participants performed statistically better than chance on the grammatical items (*M* = 60.67%, *t* = 4.00, *p* = 0.001, *d* = 1.03) and the within-modal violation items (*M* = 60.00%, *t* = 2.70, *p* = 0.017, *d* = 0.70) but not on the cross-modal violation items (*M* = 48.67%, *t* = –0.31, *p* = 0.764).

3 As in Experiment 1, all analyses were re-run after removing two problematic test items (#25 and #26) in the within-modal condition. The overall pattern of results was similar as before: the results of the ANOVA resulted in the same significant effects: Sequence type [*F*(2,29) = 13.96, *p* = 0.000, $\eta_{p}^{2}$ = 0.194] and Group [*F*(1,30) = 5.322, *p* = 0.028, $\eta_{p}^{2}$ = 0.151]. In the auditory condition, participants scored significantly better than chance when responding to grammatical (*M* = 60.63%, *t* = 3.60, *p* = 0.003, *d* = 0.90) and within-modal ungrammatical items (*M* = 79.69%, *t* = 5.69, *p* = 0.000, *d* = 0.89), but not cross-modal ungrammatical items (*M* = 50.63%, *t* = 0.13, *p* = 0.90, *d* = 0.03). In the visual condition, participants did not show scores significantly better than chance for any of the three sequence types: grammatical (*M* = 52.81%), within-modal ungrammatical (*M* = 59.38%), or cross-modal ungrammatical (*M* = 51.87%), although numerically the same overall trend is observed with highest performance on the within-modal ungrammatical items.
