# Supplementary material for: Cross-Domain Statistical–Sequential Dependencies Are Difficult to Learn
Source: Front Psychol. 2016 Feb 25;7:250. doi: 10.3389/fpsyg.2016.00250 (PMC4766371; doi:10.3389/fpsyg.2016.00250)
Supplement: Supplementary file 1 [file Appendix.DOCX]

Appendix

*Table A1.* Test items used in Experiments 1 and 2. Asterisks denote ungrammatical transitions.

| Item | Type | Stim. 1 | Stim. 2 | Stim. 3 | Stim. 4 | Stim. 5 | Stim. 6 |
| --- | --- | --- | --- | --- | --- | --- | --- |
| 1 | Grammatical | V2 | A3 | V3 | A1 | V1 | V2 |
| 2 | Grammatical | V2 | A3 | A1 | V1 | V2 | A3 |
| 3 | Grammatical | V2 | V3 | A1 | V1 | A2 | A3 |
| 4 | Grammatical | A1 | A2 | V2 | V3 | A1 | V1 |
| 5 | Grammatical | A3 | V3 | V1 | A2 | V2 | V3 |
| 6 | Grammatical | V1 | A2 | A3 | V3 | V1 | A2 |
| 7 | Grammatical | A3 | A1 | A2 | V2 | A3 | V3 |
| 8 | Grammatical | V2 | V3 | A1 | A2 | V2 | A3 |
| 9 | Grammatical | A3 | V3 | A1 | A2 | V2 | V3 |
| 10 | Grammatical | A1 | V1 | V2 | A3 | V3 | A1 |
| 11 | Grammatical | V3 | V1 | V2 | A3 | V3 | V1 |
| 12 | Grammatical | V1 | A2 | A3 | V3 | V1 | A2 |
| 13 | Grammatical | A3 | V3 | V1 | A2 | A3 | A1 |
| 14 | Grammatical | A3 | A1 | A2 | A3 | V3 | A1 |
| 15 | Grammatical | A3 | V3 | A1 | V1 | A2 | A3 |
| 16 | Grammatical | V3 | A1 | A2 | A3 | V3 | A1 |
| 17 | Grammatical | V3 | A1 | A2 | V2 | V3 | A1 |
| 18 | Grammatical | V3 | A1 | A2 | A3 | V3 | A1 |
| 19 | Grammatical | A1 | A2 | A3 | V3 | A1 | V1 |
| 20 | Grammatical | V1 | A2 | V2 | V3 | V1 | V2 |
| 21 | Violation- Within | V2 | A3 * | A2 | V2 | A3 | V3 |
| 22 | Violation- Within | A3 * | A2 | V2 * | V1 | A2 * | A1 |
| 23 | Violation- Within | A1 * | A3 | V3 | A1 * | A3 | V3 |
| 24 | Violation- Within | V2 * | V1 * | V3 | A1 * | A3 * | A2 |
| 25 | Violation- Within | A3 * | A3 * | A1 | V1 * | V3 | A1 |
| 26 | Violation- Within | V1 * | V1 * | V2 * | V1 * | V3 | A1 |
| 27 | Violation- Within | V1 | A2 * | A1 * | A3 * | A2 * | A1 |
| 28 | Violation- Within | V2 | A3 * | A2 * | A1 | V1 | A2 |
| 29 | Violation- Within | A1 | V1 | A2 * | A1 * | A3 * | A2 |
| 30 | Violation- Within | A3 | V3 | A1 | V1 * | V3 * | V2 |
| 31 | Violation-Cross | A3 * | V2 * | A2 | A3 * | V2 * | A2 |
| 32 | Violation-Cross | V2 | V3 | V1 * | A1 * | V3 | V1 |
| 33 | Violation-Cross | A2 | A3 * | V2 * | A2 * | V1 | V2 |
| 34 | Violation-Cross | A1 | A2 | A3 * | V2 * | A2 * | V1 |
| 35 | Violation-Cross | A1 * | V3 * | A3 | A1 * | V3 | V1 |
| 36 | Violation-Cross | V2 | V3 | V1 | V2 * | A2 | A3 |
| 37 | Violation-Cross | V3 * | A3 | A1 | A2 | A3 * | V2 |
| 38 | Violation-Cross | A2 * | V1 * | A1 | A2 * | V1 | V2 |
| 39 | Violation-Cross | V3 * | A2 * | V1 | V2 | V3 * | A3 |
| 40 | Violation-Cross | V3 | V1 | V2 | V3 | V1 * | A1 |
